# Development of a Direct Non-Puncture Device for Measuring Portal Venous Pressure during Liver Transplantation—A Swine Model

**DOI:** 10.3390/bios13121007

**Published:** 2023-11-30

**Authors:** Kung-Chen Ho, Tun-Sung Huang, Jiunn-Chang Lin, Huihua Kenny Chiang

**Affiliations:** 1 Department of Biomedical Engineering, National Yang-Ming Chiao-Tung University, Taipei 112, Taiwan; kungchen.6295.y@nycu.edu.tw; 2 Division of General Surgery, Department of Surgery, Mackay Memorial Hospital, Taipei 104, Taiwan; tshuang@mmh.org.tw (T.-S.H.); jiunn@mmh.org.tw (J.-C.L.); 3 Liver Medical Center, MacKay Memorial Hospital, Taipei 104, Taiwan; 4 Department of Medicine, MacKay Medical College, New Taipei City 25245, Taiwan; 5 MacKay Junior College of Medicine, Nursing, and Management, New Taipei City 11260, Taiwan

**Keywords:** liver transplantation, portal vein pressure, non-puncture PVP measuring device, swine

## Abstract

Portal hypertension-related complications pose a significant risk for liver failure post-transplantation. Thus, accurate monitoring of intraoperative portal venous pressure (PVP) is crucial. However, current PVP monitoring techniques requiring direct percutaneous puncture carry the risk of graft damage. In this study, we present an innovative non-puncture PVP monitoring device (PVPMD) using a 3D-printed prototype. PVPMD design is inspired by the sphygmomanometer principle, and strategically encompasses the portal vein and enables precise PVP measurement through blood flow ultrasonography after temporary occlusion. By a series of mini-pig experiments, the prototype PVPMD demonstrated a strong correlation with invasive catheter measurements in the main trunk of the portal vein (*r_s_* = 0.923, *p* = 0.000). There was a significant repeatability and reproducibility between the prototype PVPMD- and invasive catheter-measured PVP. This indicates that the PVPMD holds immense potential for direct application in liver transplantation and surgery. Moreover, it has the potential to replace catheter-based central venous pressure (CVP) measurements, thereby mitigating catheter-related complications during many surgeries. In conclusion, our innovative device represents a significant advancement in PVP monitoring during liver transplantation, with comprehensive validation from principle exploration to successful animal experiments. We anticipate that this groundbreaking PVPMD will attract the attention of researchers and clinicians, propelling the noninvasive measurement of PVP or other venous/arterial pressures into a new era of clinical practice.

## 1. Introduction

Liver transplantation plays an important role in the treatment of liver diseases [1] and is a life-preserving treatment for patients with end-stage hepatic failure [2]. The success of living-donor liver transplantation mostly depends on perfusion of the portal vein [3]; however, portal hypertension-related complications may lead to liver failure in the early post-transplantation period [4,5]. It is therefore critically important to have accurate and timely evidence of intraoperative portal hypertension, so that surgeons can immediately initiate hypotensive treatment, such as vasodilator therapy, portosystemic shunting, or splenectomy [4,5].

The hepatic venous pressure gradient (HVPG) has been considered the gold standard for the evaluation of portal hypertension; normal HVPG values in humans range between 1 and 5 mmHg [6]. Currently, there are two accepted methods (direct and indirect) for acquiring HVPG. Matsushima et al. described the measurement of portal venous pressure (PVP), in which the portal vein is punctured using a 23-gauge needle [7], whereas HVPG is defined as the PVP minus the central venous pressure (CVP). However, the direct puncture of the portal vein may cause serious graft damage [7] and is risky in patients with coagulation problems [8].

In contrast, the indirect method uses a balloon catheter to determine hepatic venous pressure (free hepatic venous pressure [FHVP] and wedge hepatic venous pressure [WHVP]) [9]. FHVP is measured by maintaining the tip of the “free” catheter in the right hepatic vein, resulting in a pressure that is almost equal to that of the CVP [10], whereas WHVP is measured when venous blood flow is completely blocked by the inflated balloon [11,12]. WHVP physiologically represents the sinusoidal pressure of the liver and is strongly correlated with PVP [13,14]. Accordingly, the HVPG is equivalent to the WHVP minus the FHVP, and PVP minus the CVP. However, indirect measurements of the HVPG are contraindicated in patients with severe cardiopulmonary disease, encephalopathy, or hypersensitivity to contrast dyes. Moreover, the indirect method used for HVPG measurements is associated with high technical difficulty, is expensive, and cannot provide the actual PVP [15].

Nevertheless, although the above information indicates that the PVP does not directly reflect portal hypertension, PVP is a very important parameter in HVPG. The direct method of assessing HVPG is most appropriate for liver transplantation, as PVP needs to be measured directly before and after anastomosis of the portal vein. In order to address the issues surrounding the direct measurement of PVP, we adapted the concept of a sphygmomanometer to develop a custom-made adjustable bag-based device that directly measures PVP without puncturing the vessel during liver transplantation. This study describes the validation of this portal venous pressure monitoring device (PVPMD) in animal experiments and compares the statistical correlations and agreements between the PVP values obtained by our PVPMD and those obtained using a catheter.

## 2. Materials and Methods

In this study, we developed an innovative non-puncture PVPMD using a 3D-printed prototype. A sphygmomanometer blocks the blood flow of an artery using an inflatable cuff and detects Korotkoff sounds using a stethoscope to obtain systolic and diastolic blood pressures [16]. We applied this principle to develop the PVPMD and effectively measured the relatively smooth pressure in the portal vein [17].

### 2.1. Device Design

**Figure 1:**
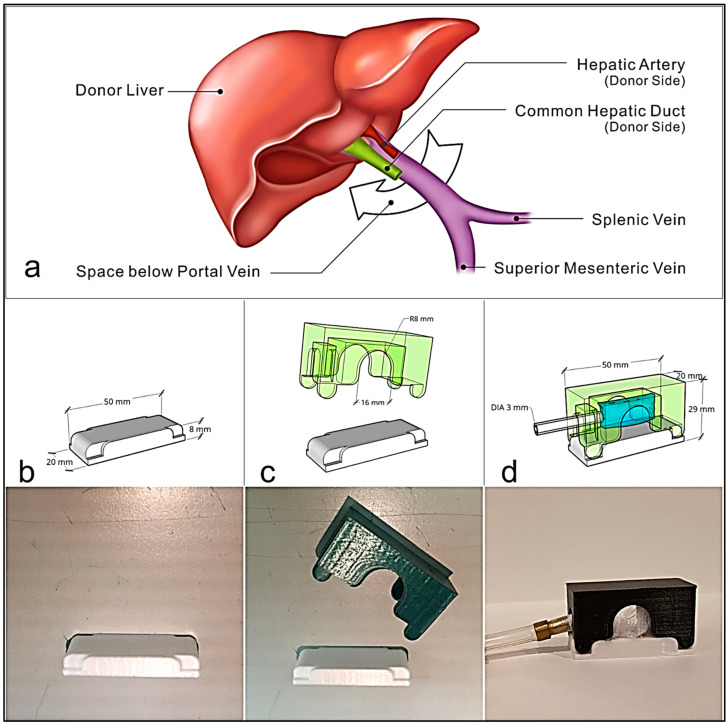
(**a**) Illustration of the surgical anatomy during liver transplantation. We used this space (white arrow under the portal vein) to design the corresponding device. (**b**–**d**) Simulation of the prototype PVPMD using 3D modeling software, with the corresponding 3D–printed components produced by a 3D Printer UP Mini 2. (**b**) The support base of the PVPMD can be inserted into the space beneath the portal vein; (**c**) The plastic portion of the adjustable bag sleeve was mounted on the support base; and (**d**) the adjustable bag sleeve can be inflated and deflated via an outer plastic tube (black arrow).

**Figure 2:**
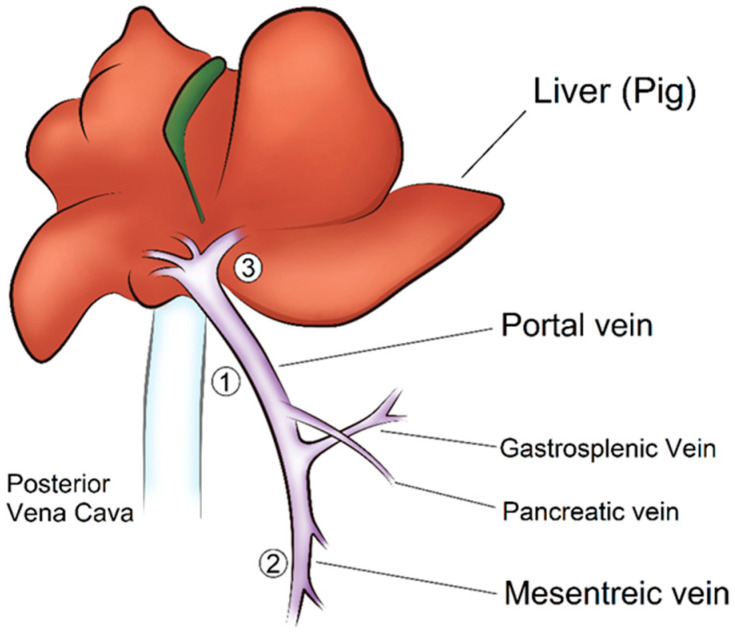
In the hepatic portal system, blood from the capillary beds of the small and large intestines, spleen, pancreas, and stomach is diverted to the liver by the hepatic portal vein before entering the posterior vena cava and returning to the heart. This anatomical drawing was a modified version of an image from the 3rd edition of *A Dissection Guide Atlas to the Fetal Pig* [21].

Figure 1a illustrates the anatomy of the operative field of the liver hilum. The distal end of the portal vein is linked to the gastrointestinal organ, whereas the proximal end is linked to the liver. We were confined to the space left below the portal vein for designing the corresponding device. Figure 1b–d show the prototype PVPMD designed using online 3D modeling software (Sketchup 2016) and created with a thermoplastic polyester-polylactide using a 3D printer (UP Mini 2, Tiertime, Williamsburg, VA, USA). We designed an arch structure that allows the portal vein to cross over the adjustable bag sleeve. The support base (thermoplastic polyester-polylactide) of the PVPMD was inserted into the space beneath the portal vein (Figure 1b), and an adjustable bag sleeve (thermoplastic polyester-polylactide) was mounted onto the support base (Figure 1c), to avoid damaging the peripheral anatomical structure. The adjustable bag sleeve was inflated and deflated using an external plastic tube, which was used to measure the pressure inside the bag (Figure 1d). The diameter of the portal vein is typically about 1.1 cm [18] and up to 2.1 cm in patients with cirrhosis and portal hypertension [19]. Based on these anatomical considerations, the inner diameter of the adjustable bag jacket has a reserved space of 1.6 cm (Figure 2, Site ①). The adjustable bag was constructed from high-density polyethylene, due to the low tensile strength and high biocompatibility of this material [20].

### 2.2. System and Data Acquisition

**Figure 3:**
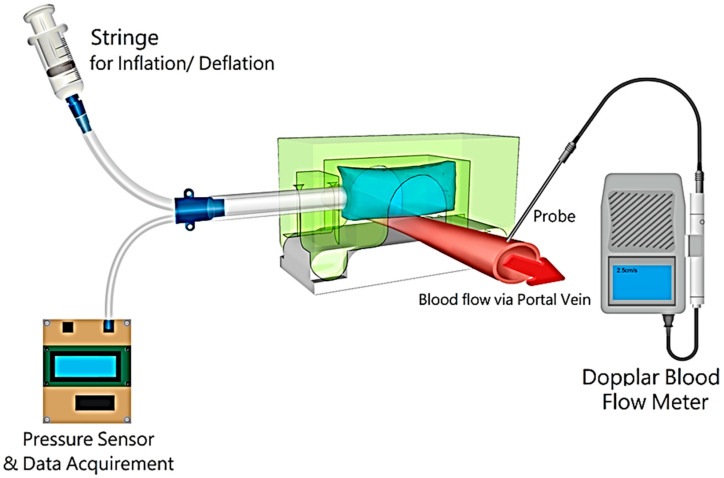
System overview. A medical syringe was used to inflate and deflate the bag of the PVPMD via an inflation tube. The pressure detector with a pressure detection panel was connected to a small plastic tube that branched off the inflation tube. The ultrasound probe of the Doppler flow probe, was placed in direct contact with the portal vein. The blood flow signal can be read on the screen of the flowmeter, which emits an audible signal in response to the blood flow.

Figure 3 schematically illustrates the cuff-like placement of the PVPMD around the portal vein. A medical syringe (inflator) was used to inflate the bag, and the volume calibration of the syringe indicated the volume of gas injected into the bag. The inflation tube and bag were connected to a pressure detector, that displayed the pressure inside the bag on the LED panel.

Our PVPMD uses a Doppler flow probe (Hadeco Cardiovascular Blood Flow Meter; Bidop^®^ ES–100V3, Kanagawa, Japan) to monitor the portal vein blood flow. This type of Doppler flow probe is commonly used in peripheral vessels. The long thin metal probe of the Doppler flow probe can be placed in direct contact with the vessel wall. The blood flow signal was read from the screen of the Doppler flow probe.

In this study, the PVPMD was placed in the portal vein, the syringe was connected to the inflation tube connected to the bag, and the Doppler flow probe was placed in contact with the wall of the portal vein. The syringe was slowly pushed to inflate the bag and the Doppler flow probe signal became weaker as the bag progressively compressed the portal vein. When the inflated bag completely blocked the blood flow, the Doppler signal disappeared from the display and the audible signal stopped. The syringe was then slowly released to reduce the pressure of the bag until the blood flow signal reappeared. The bag pressure (measured by a pressure detector) is equal to PVP when the portal vein blood flow signal and Doppler signal reappeared, and this process can be repeated several times to obtain a series of PVP values.

### 2.3. Animal Experiments

#### 2.3.1. Ethical Approval

This study was performed in line with the principles of the Declaration of Helsinki. Moreover, it also performed in the Laboratory for Animal Science of Mackay Memorial Hospital with approval from the Hospital’s Institutional Animal Care and Use Committee (Affidavit of Approval of Animal Use Protocol: MMH–A–S–109–13–R). The animals were housed and cared for in compliance with the Animal Protection Act of Taiwan.

#### 2.3.2. Preoperative Care

Three female (the anatomical structure did not interfere with the surgical field of view for liver surgery) Landrace minipigs (mean age 98 ± 14 days; mean ± standard deviation [SD] weight, 45.67 ± 1.15 kg) were fasted for 24 h before surgery with free access to water and premedicated with an intramuscular injection of 4.4 mg/kg Zoletil (Virbec, Carros, France). Endotracheal intubation was performed after fixing the animals on the experimental bed. Isoflurane anesthesia was maintained at an expiratory concentration of 1.5–4 vol%. The throat and neck were prepared and a central venous catheter (ARROWgard Blue^®^ Catheter, 7Fr Silicon catheter, Teleflex, Wayne, PA, USA) was placed in the right internal jugular vein to provide fluid resuscitation and measure CVP values. Reference CVP values for monitoring the vital signs in pigs have been published previously [22,23].

#### 2.3.3. Surgical Procedure

After a midline abdominal incision, the abdominal cavity was opened, and we explored the portal vein of the hepatic hilum. The pancreas was mobilized, and the portal vein branches were dissected. The tip of the catheter was placed in a different branch of the portal vein in each minipig, as shown in Figure 2 [21]; in the main trunk of the portal vein (site ①) in the first minipig, in the mesenteric vein (site ②) in the second minipig, and in the intrahepatic part of the portal vein (gastrosplenic vein) (site ③) in the third minipig.

After positioning the catheter, we exposed the portal vein and the PVPMD was placed at the same position on the main trunk of the portal vein, and in all animals (site ①; Figure 2). The support base was inserted into the space beneath the portal vein, and an adjustable bag sleeve was mounted on the support base. The Doppler flow probe was placed on the wall of the portal vein close to the PVPMD.

### 2.4. Data Acquisition

As shown in Figure 3, the syringe was inflated, and the bag pressure was recorded when the Doppler flow probe signal indicated that the blood flow was blocked, and the catheter pressure reading was recorded simultaneously. PVP measurements were conducted during anesthesia, and the CVP was stably maintained between 4 and 5 mmHg. CVP reflects physiological stability, and a central venous catheter can also be used for intravenous infusion [22,23,24].

### 2.5. Statistical Analysis

The nonparametric Spearman’s correlation method was used to assess correlations between PVP measurements obtained using the non-puncture PVPMD and an invasive catheter [25]. Scatter plots were used to visualize the data of Spearman’s correlation; PVPMD values were plotted on the *x*-axis and the catheter values on the *y*-axis. To evaluate whether the values determined using the PVPMD could replace catheter measurements of PVP in the clinical setting, Bland-Altman plots were used to analyze the agreement between the different methods [26]; high agreement would indicate that the PVPMD could replace catheter measurements of PVP in clinical practice. Bland-Altman plots were used to determine whether the differences between the two sets of measurements were within the eligible range [27]. The difference between the pressure values of the two methods (i.e., PVPMD-catheter) is plotted on the *x*-axis, whereas the mean of the two values [i.e., (PVPMD + catheter)/2] is plotted on the *y*-axis. All statistical analyses were performed using IBM SPSS Statistics 23 (SPSS, Chicago, IL, USA), and the plots were generated using MedCalc version 22 (MedCalc Software Ltd., Acacialaan, Belgium).

## 3. Results

### 3.1. Assessment of PVP by PVPMD and Catheter

**Figure 4:**
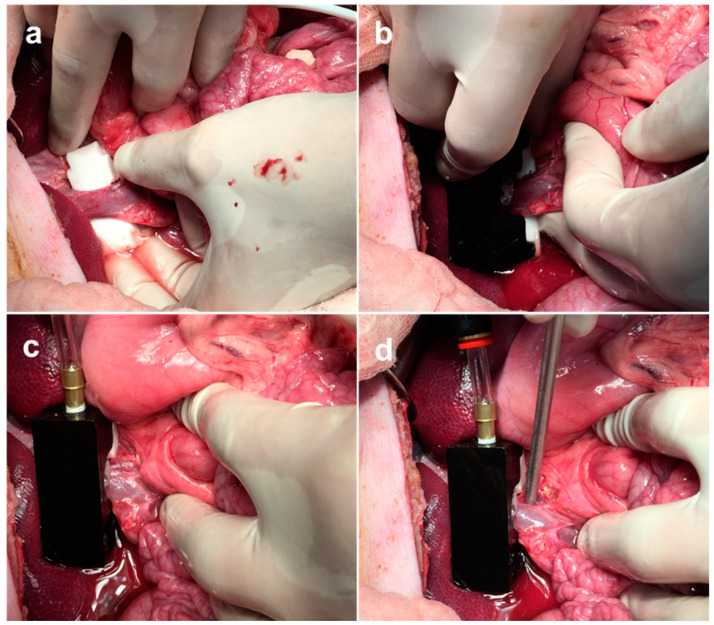
Photographs showing the use of the PVPMD during surgery. (**a**) The support base of the PVPMD is inserted into the space under the portal vein. In (**b**,**c**), an adjustable bag sleeve is mounted onto the support base. (**d**) During the measurements, the Doppler flow probe was placed in contact with the portal vein close to the device to assist in the measurement of the PVP.

As shown in Figure 4, the PVPMD was placed on the main trunk of the portal vein in a series of three PVP measuring experiments, whereas the pressure–measuring catheter was placed in the main trunk (Experiment 1), mesenteric vein (Experiment 2), or intrahepatic part of the portal venous system (Experiment 3). In the three experiments, the weights of the minipigs were 44.5, 46.8 and 45.7 kg, respectively (Table 1). The mean CVP were 4.4 ± 0.7, 4.7 ± 0.7 and 4.8 ± 0.5, respectively (Table 1).

**Figure 5:**
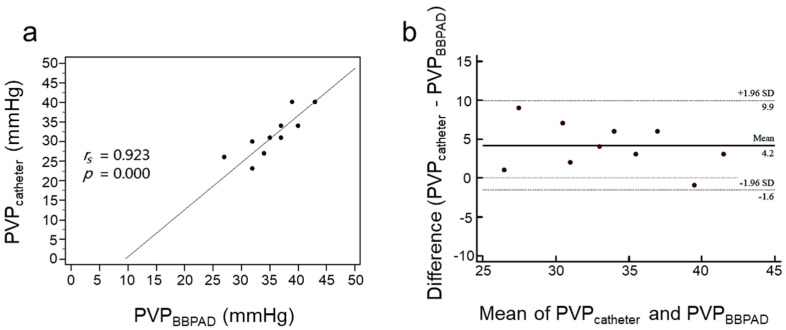
Correlations and agreements between the pressure values obtained using the PVPMD and the catheter were placed on the main trunk of the portal vein. (**a**) Spearman’s correlation (**b**) Bland–Altman agreement.

Figure 5 shows the measurement of the PVP by placing both the PVPMD and the pressure catheter in the main trunk of the portal vein (1st PVP experiment), the mean PVP obtained using the PVPMD was 35.7 ± 4.4 mmHg and the mean PVP measured using the catheter was 31.5 ± 5.3 mmHg (Table 1). Spearman’s correlation analysis showed a significant correlation between the two measurements (*r_s_* = 0.923, *p* = 0.000; Figure 5a), indicating a strong correlation [28,29]. In the Bland–Altman plot, all of the data points obtained using the PVPMD and catheter were within the 95% confidence interval (CI). Most points showed horizontal zonation; good agreement was achieved with a mean difference of 4.2 mmHg and a standard deviation of 2.93 mmHg, with 95% limits of agreement (LoA) ranging from −1.6 to 9.9 (Figure 5b).

**Figure 6:**
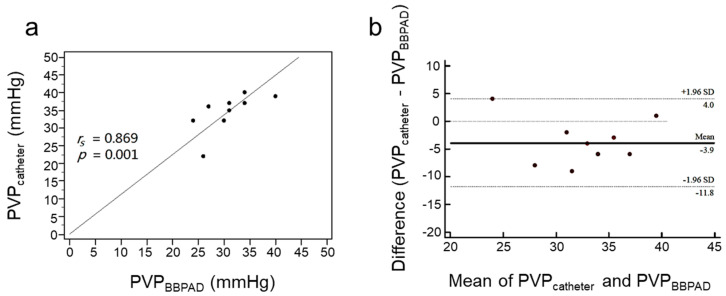
Correlations and agreements between the pressure values obtained using the PVPMD were placed on the main trunk of the portal vein and a catheter located in the distal mesenteric vein. (**a**) Spearman’s correlation. (**b**) Bland–Altman agreement.

Figure 6 showed the 2nd PVP experiment, the mean PVP measured using the PVPMD on the main trunk of the portal vein was 30.80 ± 4.6 mmHg and the mean PVP obtained using the pressure catheter in the distal mesenteric vein was 34.7 ± 5.2 mmHg. Spearman’s correlation analysis showed a strong correlation between the two measurements (*r_s_* = 0.869, *p* = 0.001) (Figure 6a) [28,30]. In the Bland-Altman plot, the mean difference was −3.9 mmHg with a standard deviation of 4.04 mmHg and the CI of the 95% LoA ranged from −11.8 to 4.0; 90% of the points were within the 95% confidence range and most data points were close to the horizontal area, indicating good agreement (Figure 6b).

**Figure 7:**
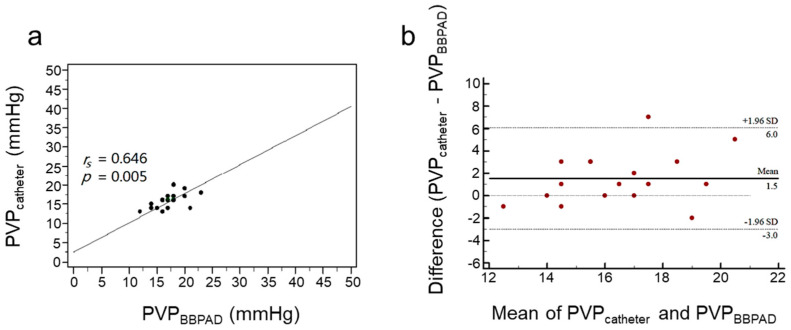
Correlations and agreements between the pressure values obtained using the PVPMD were placed on the main trunk of the portal vein and a catheter located in the intrahepatic part of the portal vein. (**a**) Spearman’s correlation. (**b**) Bland–Altman agreement.

Figure 7 shows the 3rd experiment, the mean PVP measured using the PVPMD on the main trunk of the portal vein was 17.2 ± 2.7 mmHg and the mean PVP obtained using the pressure catheter in the intrahepatic part of the portal vein (gastrosplenic vein) was 15.7 ± 2.1 mmHg. Spearman’s correlation analysis revealed a moderate correlation (*r_s_* = 0.646, *p* = 0.005; Figure 7a) [28,29]. In the Bland-Altman plot, the mean difference was 1.5 mmHg with a standard deviation of 2.29 mmHg and the CI of 95% LoA ranged from −3.0 to 6.0 (Figure 7b). Overall, 94.1% of the points in the third experiment were within the 95% CI, mostly close to the horizontal zonation, indicating a good agreement (Figure 7b).

Furthermore, we also determined the consistency of the two measurements in each experiment using alternate-form reliability analysis. As shown in Supplementary Table S1, there was significant consistency between both measurements in each experiment (1st experiments: *r* = 0.901, *p* = 0.001; 2nd experiment: *r* = 0.794, *p* = 0.010; 3rd experiment: *r* = 0.716, *p* = 0.008).

### 3.2. Repeatability and Reproducibility of the PVPMD-Measured PVP

We further analyzed the consistency of the two measurements across the different experiments using alternate-form retest reliability analysis. As shown in Supplementary Table S1, there was a significant consistency between the two measurements across the three experiments (*r* = 0.945, *p* < 0.001). This indicates that PVPMD provides a good repeatability and reproducibility for measuring PVP in the different pigs.

## 4. Discussion

Accurate measurement of the PVP during liver transplantation is critical for ensuring successful outcomes. Hepatic vein catheterization and measurement of HVPG are the gold standards for this purpose and correlate well with portal pressure. However, current methods have limitations, including the risk of vascular and graft damage with direct catheterization and the indirect determination of the PVP with noninvasive methods that do not provide accurate values [7,8,31,32,33]. In this respect, PVPMD can obviate many of these limitations because the procedure is direct, simple, and noninvasive. In the present study, we introduced a novel sphygmomanometer principle-based device, the PVPMD, that provides accurate and consistent intraoperative PVP measurements in liver transplants. Our results demonstrated that the PVPMD offers a reliable alternative to direct catheterization, with the added benefits of being noninvasive and easy to use. Using the PVPMD, liver transplantation teams can obtain real-time PVP measurements during surgery, which can help guide surgical decisions and improve patient outcomes. This study represents a significant advancement in the field of liver transplantation and highlights the potential of the PVPMD to revolutionize PVP monitoring.

In the present study, our noninvasive direct method for measuring PVP was based on the principle of Bernoulli’s equation, which is like the technique used to measure arterial pressure using a sphygmomanometer. For traditional blood pressure readings, systolic and diastolic pressures must be guided by Korotkoff sounds using a stethoscope [17]. By applying this principle to measure the relatively smooth pressure in the portal vein [32], our method avoids vascular damage. PVPMD is structurally compatible with the pitot tube concept [34]. The bag of the PVPMD was slowly inflated to compress the portal vein until the flow was blocked. We inflated the bag until the bag pressure was just high enough to block the blood flow (P_Bag_); at this point, the flow velocity was zero, the Doppler signal disappeared from the display, and the audible signal stopped. The syringe was then slowly released to reduce the pressure of the bag until the blood flow signal reappeared. As shown by fluid mechanics (Bernoulli equation), in order to reduce the flow rate to zero, lateral vascular compression must be performed at least equal to the vascular pressure. The bag pressure is equal to that of PVP when the portal vein blood flow and Doppler signals reappear. In the in vitro study, we also proposed a fluid mechanics proof-of-concept for PVPMD that directly and accurately measured the PVP.

We compared the PVP measurements obtained using the PVPMD placed on the main trunk of the portal vein and the pressure catheter placed in the mesenteric or the intrahepatic part of the portal vein. We observed the highest correlation and consistency of the PVP measurements obtained using both the PVPMD and the pressure catheter placed on the main trunk of the portal vein (Figure 5, Figure 6 and Figure 7). This indicates that there is a difference between the catheter measurements of the main trunk pressure and branch pressure, with slightly inaccurate catheter measurements of the branch pressure. The trunk pressure was measured using the PVPMD, i.e., the actual PVP. In contrast, when the catheter was placed in the branches of the portal venous system and the PVPMD was placed on the main trunk, in accordance with the principles of the continuous equation of fluid mechanics in parallel pipes, the values measured by the catheter in the branches of the portal system and by the PVPMD cannot be equal [24,35]. This concept also explains the strong and statistically significant correlation between PVPMD and catheter placed in the main trunk of the portal vein (*r_s_* = 0.923, *p* = 0.000). Notably, in previous studies of liver transplantation, direct puncture of the main trunk of the portal vein has rarely been performed to measure PVP [17,26]. Although direct puncture of the vessel just after portal vein anastomosis has been performed during transplantation, the resulting damage is unpredictable. Therefore, most relevant studies have used branches of the portal vein system, such as the superior mesenteric [36], inferior mesenteric [37], splenic [31], jejunal, or ileal mesenteric veins [38]. However, the PVPMD proposed in this study can be used to accurately and safely measure the actual PVP without needing to puncture the vessel.

In the present study, we observed a significant correlation between PVP measurements obtained using the PVPMD on the main trunk of the portal vein and an invasive catheter placed on the main trunk of the portal vein, the mesenteric vein, or intrahepatic part of the portal vein. This suggests that our new method can achieve accurate PVP measurement. Nevertheless, when the PVPMD and catheter were placed on the main trunk of the portal vein to determine PVP, we observed a slightly higher PVP obtained with the PVPMD than with the catheter-determined values. For this aspect, it might be caused by a delayed operator response. The operator may manually continue inflating the bag for a short time after the Doppler signal disappears, which would lead to a slightly higher PVPMD pressure reading. This phenomenon can be improved by designing an automatic control PVPMD system or integrating a Doppler sensor-based PVPMD system in the future.

We also evaluated the consistency of the PVP measurements across different experiments using alternate-form retest reliability analysis. The results demonstrated significant consistency between the two measurements across the three experiments. Moreover, the coefficient of consistency between these two measurements across the experiments was higher than that for each individual experiment. This suggests that our device has an accurate alternate-form retest reliability for measuring PVP in different experiments, even though the catheter was placed in a different part of the portal venous system in each experiment.

In recent decades, liver surgery has undergone important changes, although the surgical management of cirrhotic liver remnants remains problematic [39]. The liver could be regenerated, allowing for repeated resections. In some cases, when this ability is impaired, or when an extensive resection of small liver remnants is performed, these patients may develop small-for-size syndrome, in which the reduced liver mass may be inadequate for maintaining normal liver function. Notably, preoperative portal pressure is an important predictor of hepatic decompensation in cirrhotic liver after resection for hepatocellular carcinoma (HCC). As assessed by Bruix et al. [40], HVPG (PVP) was significantly associated with unresolved decompensation within 3 months after surgery, with an odds ratio of 1.9. Hidaka et al. [41] have also shown that high portal pressure is associated with poor long-term prognosis after hepatectomy in HCC. According to these findings, we recommend that intraoperative measurement of PVP using the PVPMD may be a potential strategy for guiding the quantity of hepatic tissue removal in patients with liver cirrhosis who undergo liver resection. Our cost–effective procedure may also provide noninvasive and direct measurements of PVP. Certainly, our method merits further investigation.

The development of massive ascites is a known complication after liver resection, which increases the risk of further complications, such as post-hepatectomy liver failure (PHLF) [42]. Although frequently encountered in the postoperative setting, the underlying pathophysiology of these complications is not yet fully understood. Nevertheless, the sudden changes in liver macroscopic hemodynamics may be closely related to the development of ascites and related complications [43,44]. In particular, the high intravascular shear stress associated with acute portal vein hyperperfusion leads to liver dysfunction after extended resection [45]. Bogner et al. [46] have revealed that an intraoperative increase in PVP is an independent predictor of PHLF. Accordingly, we recommend routine intraoperative monitoring of PVP in patients who undergo a liver resection, to prevent the occurrence of these complications. Our PVPMD may potentially be used for this intraoperative monitoring, as it provides noninvasive and direct measurements of portal venous pressure.

### Limitation and Future Outlook

The main limitations of our study arise from its methodology, as it was a swine model with a relatively small sample size, although PVPMD provided a good repeatability and reproducibility for measuring PVP in the different pigs. Moreover, further investigation should be addressed to perform postoperative evaluation in the pigs because the safety and biocompatibility are important to the medical device, although the design of the PVPMD had to avoid the damage of peripheral anatomical structure of portal vein. In addition, we focused on validating the concept and feasibility of the PVPMD in a simple animal model. However, for clinical application in human liver transplantation, further development is needed to optimize the device design and ensure compatibility with all steps of the procedure, including arterial anastomosis and biliary anastomosis. It is important that the device does not obstruct the surgical process or hinder access to the necessary anatomical structures. Furthermore, in future iterations of the PVPMD, embedding a space for the Ultrasound Doppler flow sensor within the PVPMD could further enhance the functionality and accuracy of the device. These advancements can increase the clinical utility of the PVPMD and make it a valuable tool for real-time measurement of PVP during liver transplantation. Further clinical validation and development are needed to refine the device and address the specific challenges associated with its application in human patients undergoing liver transplantation.

## 5. Conclusions

In conclusion, we demonstrated the feasibility of using a custom-made PVPMD combined with a Doppler flow probe to accurately measure portal venous pressure in a simple animal model. Further development is needed to design an optimal device for use in human liver transplantation, including arterial and biliary anastomosis. Incorporating and embedding the Doppler flow probe in the PVPMD could enhance the device’s functionality and accuracy. The non-puncture PVPMD offers a safe and reliable method for measuring PVP on the main trunk of the portal vein, providing more accurate measurements compared to conventional branch pressure measurements. This device has the potential to greatly improve the monitoring and management of PVP during liver transplantation, ultimately preventing complications and optimizing patient outcomes.

## Figures and Tables

**Table 1:** The weight of the animals and PVP values obtained from the PVPMD were placed on the main trunk of the portal vein, and a catheter was placed at three different positions on the branches of the portal vein system.

	1st Experiment	2nd Experiment	3rd Experiment
Weight (kg)	44.5	46.8	45.7
Number of measurements (*n*)	11	10	17
Central venous pressure (mmHg), mean ± SD	4.4 ± 0.7	4.7 ± 0.7	4.8 ± 0.5
Portal pressure via PVPMD (mmHg), mean ± SD	35.7 ± 4.4	30.8 ± 4.68	17.2 ± 2.7
Catheter site	Main trunk	Mesenteric vein	Intrahepatic part
Portal pressure via catheter (mmHg), mean ± SD	31.5 ± 5.3	34.7 ± 5.2	15.7 ± 2.1

The Central venous pressure was maintained during the measurement process and remained between 4 and 5 mm Hg in all three experiments.

## Data Availability

The data used to support the findings of this study are included within the article and its Supplementary Information Files.

## Supplementary Materials

The following supporting information can be downloaded at: https://www.mdpi.com/article/10.3390/bios13121007/s1, Table S1. Estimates of the alternate-form coefficient and alternate-form retest coefficient for minipig experiments.
