# Supplementary material for: Development of a Direct Non-Puncture Device for Measuring Portal Venous Pressure during Liver Transplantation—A Swine Model
Source: Biosensors (Basel). 2023 Nov 30;13(12):1007. doi: 10.3390/bios13121007 (PMC10742213; doi:10.3390/bios13121007)
Supplement: Supplementary file 1 [file biosensors-13-01007-s001.zip › biosensors-2620103-supplementary.pdf]

Development of a Direct, Non-Puncture Device for Measuring Portal Venous Pressure during Liver Transplantation - a Swine Model

Supplementary Table S1. Estimates of the alternate-form and alternate-form retest coefficients for minipig experiments.

**Reliablility**	***r***	**p-value**
**Alternate-form coefficient**		
Minipig 1	0.901	0.001*
Minipig 2	0.794	0.010*
Minipig 3	0.716	0.008*
**Alternate-form retest coefficient across 3 minipigs**	0.945	<0.001*
